# Shuanghuang Shengbai granule cures myelosuppression and suppresses lung cancer progression: mechanism and therapeutic targets from the aspect of microRNAs

**DOI:** 10.18632/oncotarget.19129

**Published:** 2017-07-10

**Authors:** Shuang Wang, Zhenye Xu, Lifang Wang

**Affiliations:** ^1^ Department of Oncology II, Longhua Hospital, Shanghai University of Traditional Chinese Medicine, Shanghai 200032, China; ^2^ Department of Oncology, Seventh People’s Hospital, Shanghai University of Traditional Chinese Medicine, Shanghai 200137, China

**Keywords:** cyclophosphamide, lung cancer, myelosuppression, Shuanghuang Shengbai granule, white blood cells

## Abstract

**Background:**

Shuanghuang Shengbai granule is effective in curing cyclophosphamide-induced myelosuppression without promoting lung cancer development. This study aims to investigate its mechanism and therapeutic targets.

**Methods:**

Nude mice with lung cancer were treated with physiological saline (control), cyclophosphamide, or cyclophosphamide + Shuanghuang Shengbai. MicroRNA microarray was used to investigate the differentially expressed microRNAs in lung cancer stem cells or bone marrow hematopoietic stem cells between the three groups. MicroRNA expressions were confirmed using quantitative real time-polymerase chain reaction.

**Results:**

Cyclophosphamide suppressed tumor growth and decreased the ratio of SP^+^ lung cancer stem cells (P<0.05). Shuanghuang Shengbai further decreased the ratios of SP^+^ and CD24^+^IGF1R^+^ lung cancer stem cells (P<0.05). Shuanghuang Shengbai completely reversed the cyclophosphamide-induced decreases in white blood cells, proliferation index of bone marrow cells, and the ratio of CD34^+^SCA1^+^ bone marrow hematopoietic stem cells (P<0.05). We found 45 and 343 altered microRNAs for SP^+^ lung cancer stem cells and CD34^+^SCA1^+^ bone marrow hematopoietic stem cells, respectively. Moreover, miR-32*, miR-466i-5p, and mmu-miR-669c in SP^+^ lung cancer stem cells were confirmed, as well as mmu-miR-106b*, mmu-miR-144, mmu-miR-669k*, mmu-miR-142-3p, mmu-miR-210, and mmu-miR-223 in CD34^+^SCA1^+^ bone marrow hematopoietic stem cells.

**Conclusion:**

Shuanghuang Shengbai might promote the proliferation of CD34^+^SCA1^+^ bone marrow hematopoietic stem cells via up-regulating mmu-miR-106b*, mmu-miR-144, and mmu-miR-669k*, as well as down-regulating mmu-miR-142-3p, mmu-miR-210, and mmu-miR-223. Shuanghuang Shengbai might further inhibit the proliferation of SP^+^ lung cancer stem cells via enhancing the expressions of miR-32*, miR-466i-5p, and mmu-miR-669c. These might be the mechanism and therapeutic targets of Shuanghuang Shengbai granule.

## INTRODUCTION

Lung cancer is the most common cancer in men and the leading cause of cancer death in women [1]. The 5-year relative survival of lung cancer is currently 18% [2]. Especially, lung cancer diagnosed in China comprises about 33-50% of the global incidence burden. For lung cancer, 733,300 new cases and 432,400 deaths were estimated to appear in China in 2015 [3]. Cyclophosphamide (CTX) has been widely used to block the progression of lung cancer in clinic [4]. However, CTX generally causes severe acute toxicities like myelosuppression [5]. Once myelosuppression appears, patients are more likely to suffer from infection. Currently, protecting patients from CTX-induced myelosuppression is a main challenge in lung cancer treatment.

Nowadays, colony-stimulating factor, granulocyte colony-stimulating factor and granulocyte-macrophage colony-stimulating factor are the main drugs in treating myelosuppression [6, 7]. However, these drugs can lead to lung injury, splenic rupture and vascular events, and they even promote cancer development and metastasis [8, 9]. As a traditional Chinese medicine, Shuanghuang Shengbai (SHSB) granule has been widely used in clinic for decades in China [10]. It can increase white blood cells (WBCs) and cure myelosuppression, whereas it cannot cause obvious adverse effect or cancer progression [11, 12]. Reportedly, SHSB granule can enhance the epidermal growth factor receptor signaling pathway in bone marrow and the Notch signaling pathway in bone marrow nuclear cells [13, 14]. SHSB granule can reduce the percentage of bone marrow cells (BMCs) in G0/G1 phase and increase the proliferation index of BMCs via up-regulating CDK4, CDK6 and cyclin D1 [15]. Also, it promotes the transformation of hematopoietic stem cells from G0/G1 phase to S phase via up-regulating c-Myc, cell division cycle 25A, Rb, pRb and E2F in bone marrow nuclear cells [10]. On the contrary, it inhibits the proliferation of tumor cells in an opposite way [10, 15].

Reportedly, WBCs are mainly differentiated from bone marrow hematopoietic stem cells (BMHSCs; e.g. CD34^+^SCA1^+^ BMHSCs). Lung cancer stem cells (LCSCs) are responsible for the initiation, progression, metastasis and relapse of lung cancers (e.g. SP^+^ LCSCs, CD24^+^IGF1R^+^ LCSCs and CD133^+^ LCSCs) [16, 17]. Therefore, the proliferation and numbers of BMHSCs and LCSCs are critical for myelosuppression and lung cancer. As a kind of non-coding RNAs, microRNAs play crucial roles in cell proliferation via regulating the expression of target genes post-transcriptionally [18, 19]. However, the effect of SHSB granule on LCSCs and BMHSCs and the corresponding mechanism at microRNA level are still unclear.

In the present study, nude mice with lung cancer were treated with physiological saline, CTX, or CTX + SHSB granule. Then, LCSCs ratio and BMHSCs ratio were determined. Microarray analysis was performed to study the microRNA profiling of LCSCs and BMHSCs. MicroRNA expressions were further validated using quantitative real time-polymerase chain reaction (qRT-PCR). Our results might shed light on the mechanism and therapeutic targets of SHSB granule in curing myelosuppression from the aspect of microRNAs.

## RESULTS

### Tumor mass and general blood indexes

**Figure 1:**
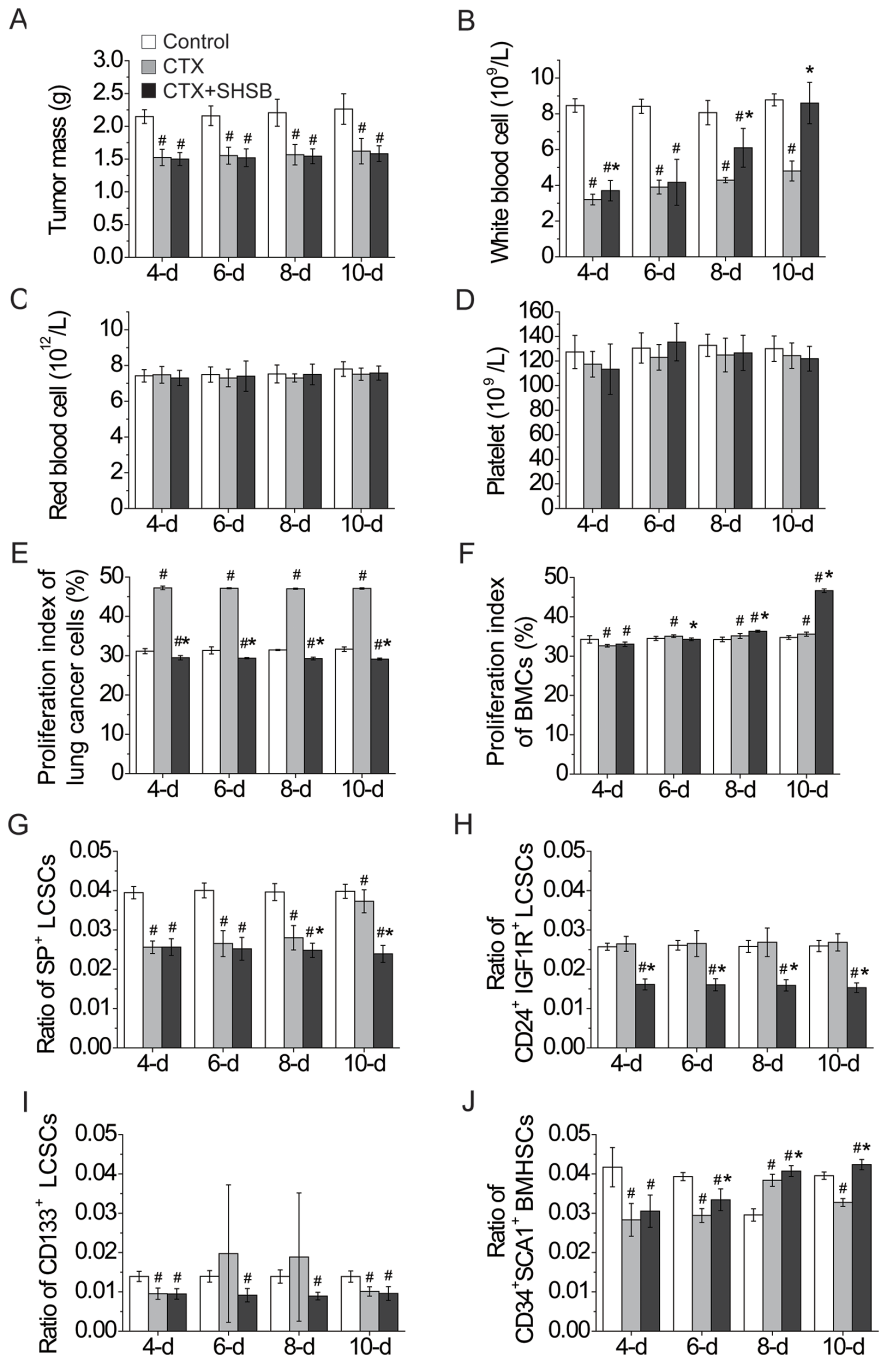
Effects of CTX and SHSB granule on nude mice with lung cancer **(A)** Tumor mass. **(B)** White blood cells. **(C)** Red blood cells. **(D)** Platelets. **(E)** Proliferation index of lung cancer cells. **(F)** Proliferation index of BMCs. **(G)** Ratio of SP^+^ LCSCs. **(H)** Ratio of CD24^+^IGF1R^+^ LCSCs. **(I)** Ratio of CD133^+^ LCSCs. **(J)** Ratio of CD34^+^SCA1^+^ BMHSCs. #: P<0.05 when compared with control group. *: P<0.05 when compared with CTX group. CTX: cyclophosphamide; SHSB: Shuanghuang Shengbai; BMCs: bone marrow cells; LCSCs: lung cancer stem cells; BMHSCs: bone marrow hematopoietic stem cells. For all these tests, N=9.

Tumor masses in CTX and CTX+SHSB groups were significantly smaller than that in control group (4-d, 6-d, 8-d and 10-d; P<0.05; Figure 1A). When compared with control group, WBCs were significantly decreased after CTX injection (4-d, 6-d and 8-d; P<0.05; Figure 1B). However, SHSB treatment reversed the CTX-induced decline in WBCs, and the number of WBCs was completely recovered 10 days after SHSB treatment (10-d; Figure 1B). Besides, red blood cells and platelets in the three groups were similar (4-d, 6-d, 8-d and 10-d; Figure 1C and 1D).

### Cell cycle and proliferation index

Proliferation index of lung cancer cells in CTX+SHSB group was significantly lower than that in CTX and control groups (4-d, 6-d, 8-d and 10-d; P<0.05; Figure 1E).

Moreover, the proliferation index of BMCs in CTX group was first significantly decreased (4-d; P<0.05) and then increased (6-d, 8-d and 10-d; P<0.05; Figure 1F) in comparison with that in control group. In contrast, the proliferation index of BMCs in CTX+SHSB group was remarkably higher than that in CTX and control groups (8-d and 10-d; P<0.05; Figure 1F).

### Ratio of stem cells

SP^+^ LCSCs ratio was significantly declined after CTX injection and further declined after CTX+SHSB treatment (8-d and 10-d; P<0.05; Figure 1G and Supplementary Figure 1). CD24^+^IGF1R^+^ LCSCs ratio in control and CTX groups were similar, whereas it was remarkably decreased after CTX+SHSB treatment (4-d, 6-d, 8-d and 10-d; P<0.05; Figure 1H and Supplementary Figure 1). In addition, CD133^+^ LCSCs ratio was declined after CTX or CTX+SHSB treatment (4-d and 10-d; P<0.05; Figure 1I and Supplementary Figure 1).

In comparison with control group, CD34^+^SCA1^+^ BMHSCs ratio in CTX group was first decreased (4-d and 6-d; P<0.05), then increased (8-d; P<0.05), and finally decreased (10-d; P<0.05) after CTX injection (Figure 1J and Supplementary Figure 1). In contrast, CD34^+^SCA1^+^ BMHSCs ratio in CTX+SHSB group was remarkably higher than that in CTX group and constantly increased along with the treating time (4-d, 6-d, 8-d and 10-d; P<0.05; Figure 1J and Supplementary Figure 1). These results were consistent with the results of proliferation index of BMCs (Figure 1F).

### MicroRNA microarray analysis

**Figure 2:**
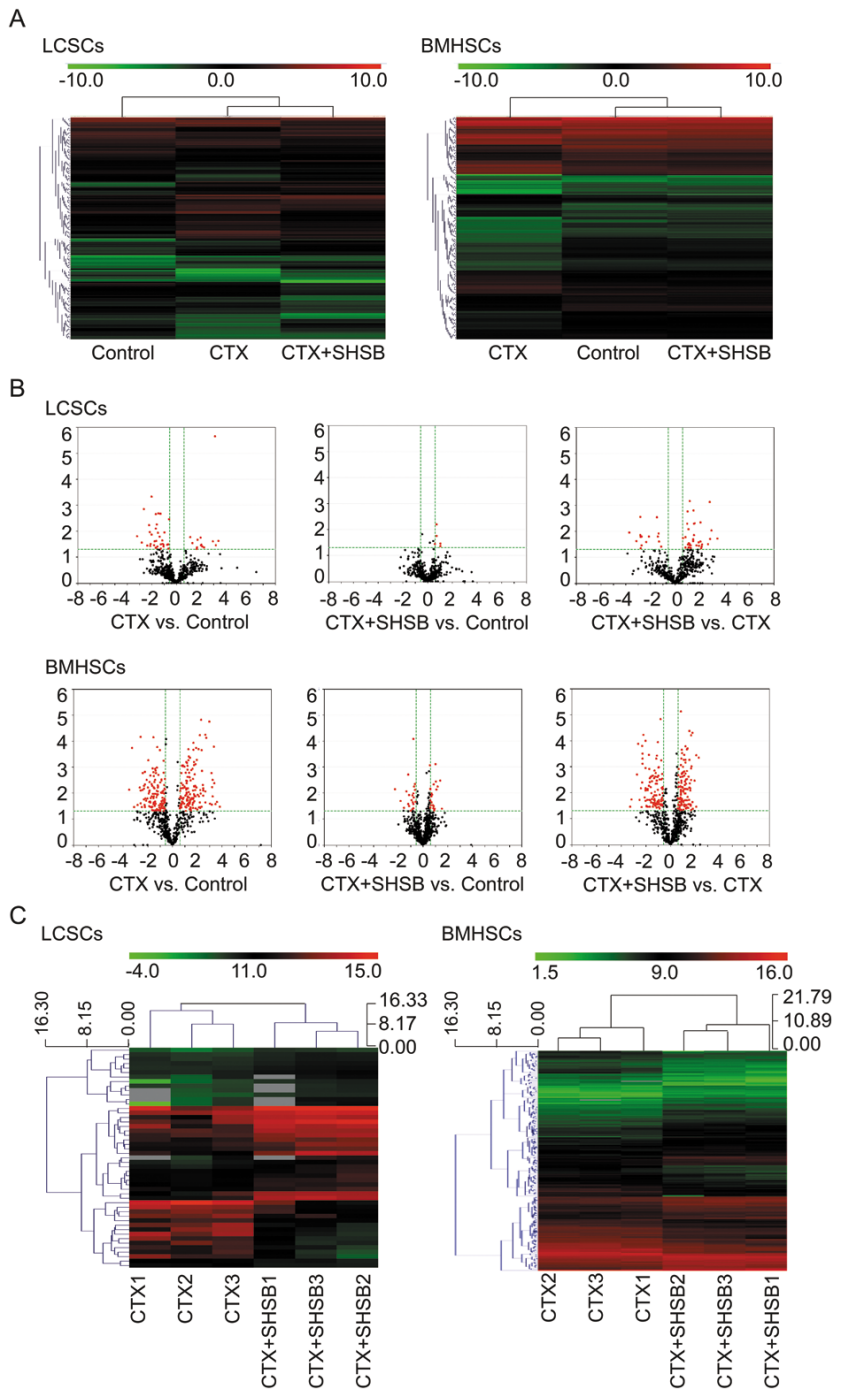
Microarray analysis of microRNAs in LCSCs and BMHSCs **(A)** Heat-maps of microRNAs in LCSCs and BMHSCs in the three groups. **(B)** Volcano plot filtering and DEmiRNAs screening. **(C)** Hierarchical clustering of DEmiRNAs between CTX and CTX+SHSB groups. LCSCs: lung cancer stem cells; BMHSCs: bone marrow hematopoietic stem cells; DEmiRNAs: differentially expressed microRNAs; CTX: cyclophosphamide; SHSB: Shuanghuang Shengbai.

MicroRNAs isolated from SP^+^ LCSCs (8-d) and CD34^+^SCA1^+^ BMHSCs (6-d) were analyzed using microarray (miRCURY™ LNA Array, version: 16.0; Figure 2A). Based on Volcano Plot filtering (Figure 2B), 49, 5 and 49 DEmiRNAs in LCSCs were found between CTX and control groups, CTX+SHSB and control groups, as well as CTX+SHSB and CTX groups, respectively (Fold change ≥1.5 and P≤0.05; Figure 2C). A total of 278, 45 and 234 DEmiRNAs in BMHSCs were identified between CTX and control groups, CTX+SHSB and control groups, as well as CTX+SHSB and CTX groups, respectively (Fold change ≥1.5 and P≤0.05; Figure 2C). Furthermore, ANOVA analysis identified 45 DEmiRNAs (in LCSCs) and 343 DEmiRNAs (in BMHSCs) between the three groups (Table 1).

**Table 1:** DEmiRNAs in LCSCs and BMHSCs

Cell	DEmiRNA				
**LCSCs**	mmu-miR-3095-3p	mmu-miR-669a/o-3p	mmu-miR-466f	mmu-miR-431	mmu-miR-3081*
	mmu-miR-466a/p-5p	mmu-miR-379	mmu-miR-1897-5p	mmu-miR-488*	mcmv-miR-M23-2*
	mmu-miR-32*	mmu-miR-3065*	mmu-miR-194-2*	mmu-miR-1192	mmu-miR-675-5p
	mmu-miR-466a-5p	**mmu-miR-466i-5p**	mmu-miR-881*	mmu-miR-106b*	mmu-miR-881
	mmu-miR-34c*	mmu-miR-3071	mmu-miR-122*	mmu-miR-1187	mmu-miR-291a-5p
	mmu-miR-3082-5p	mmu-miR-669k*	mmu-miR-669d	mmu-miR-3067	mmu-miR-669o-5p
	mmu-miR-195*	mmu-miR-669e	mmu-miR-466c-5p	mcmv-miR-m01-2	mmu-miR-669l
	mmu-miR-669f-5p	mmu-miR-3078*	mmu-miR-466b/o-5p	mmu-miR-669n	mmu-miR-541*
	**mmu-miR-669c**	mmu-miR-706	mmu-miR-466e-5p	mmu-miR-669p*	mmu-miR-221*
**BMHSCs**	mmu-miR-3103*	mmu-miR-194	mmu-miR-669l*	mmu-miR-669m-3p	mmu-miR-19a
	mmu-miR-434-3p	mmu-miR-32*	mmu-miR-192	mmu-miR-1971	mmu-miR-126-5p
	mmu-miR-467d*	mmu-miR-145*	mmu-miR-99a	mmu-miR-677	mmu-miR-215
	mmu-miR-467b*	mmu-miR-762	mmu-miR-374	mmu-miR-350	mmu-miR-681
	mmu-miR-714	mmu-miR-499	mmu-let-7c	mmu-miR-16-2*	mmu-miR-133b
	mmu-miR-466i-3p	mmu-miR-702	mmu-miR-466a/e-3p	mmu-miR-196a-1*	mmu-miR-291b-3p
	mmu-miR-883a-5p	mmu-miR-329*	mmu-miR-136	mmu-miR-675-5p	mmu-miR-1962
	mmu-miR-3090*	mmu-miR-376a	mmu-miR-466f-3p	mmu-miR-411	mmu-miR-3098-3p
	mmu-miR-669d-2*	mmu-miR-7b*	mmu-miR-142-5p	mmu-miR-434-5p	mmu-miR-146a
	mmu-miR-100	mmu-miR-224	mmu-miR-1946b	mmu-miR-1b-5p	mmu-miR-496*
	mmu-miR-466m-3p	mmu-miR-466a/b/c/e/p/n-3p	mmu-miR-3086-3p	mmu-let-7d*	mmu-miR-300
	mmu-miR-721	mmu-miR-466n-3p	mmu-miR-29a*	mmu-miR-1247	mmu-miR-345-5p
	mmu-miR-467c*	**mmu-miR-144**	mmu-miR-770-3p	mmu-miR-1a-1*	mmu-miR-465c-5p
	mmu-miR-1900	mmu-miR-2861	mmu-miR-34c*	mmu-miR-154*	mmu-miR-138-2*
	mmu-miR-3095-3p	mmu-miR-493	mmu-miR-22	mmu-miR-101a	mmu-miR-1249
	mmu-miR-23a*	mmu-miR-668*	mmu-miR-296-3p	mmu-miR-106a	mmu-miR-877
	mcmv-miR-m88-1	mmu-miR-470	mmu-miR-29b	mmu-miR-24-2*	mmu-miR-21*
	mmu-miR-3064-5p	mmu-miR-146b	mmu-miR-669h-3p	mmu-miR-185*	mmu-miR-669m/466m-5p
	mmu-miR-490-3p	mmu-miR-292-5p	mmu-miR-466l-3p	mmu-miR-1931	mmu-miR-703
	mmu-miR-24	mmu-miR-5097	mmu-miR-152	mmu-miR-325*	mmu-miR-140
	mmu-miR-467e*	mmu-miR-145	mmu-miR-290-3p	mmu-miR-344g-5p	mmu-miR-15a
	mmu-miR-127	mmu-miR-19b	mmu-miR-3104-5p	mmu-miR-128-2*	mmu-miR-328
	mmu-miR-467a*	mmu-miR-194-2*	mmu-miR-1187	mmu-miR-149	mmu-miR-378
	mmu-miR-29c	mghv-miR-M1-2-3p	mmu-miR-125a-3p	mmu-miR-706	mmu-miR-450a
	mmu-miR-297a*/c*/-297b-3p	mmu-miR-92a-2*	mmu-miR-132*	mmu-miR-1194	mmu-miR-339-5p
	mmu-miR-138-1*	mmu-miR-218	mmu-miR-224*	mmu-miR-365-2*	mmu-miR-1949
	mghv-miR-M1-5	mmu-miR-669i	mmu-miR-503*	mghv-mir-M1-1*	**mmu-miR-142-3p**
	mmu-miR-466g	mmu-miR-3057-5p	mmu-miR-216a*	mmu-miR-582-3p	mmu-miR-300*
	mmu-miR-27b	mmu-miR-574-3p	mmu-miR-290-5p	mmu-miR-3060*	mmu-let-7e
	mmu-miR-669d*/669d-2*	mmu-miR-3097-5p	mmu-miR-678	mmu-miR-2139	mmu-miR-503
	mmu-miR-195*	mmu-miR-3078*	mmu-miR-3073-3p	mmu-miR-30c-2*	mmu-miR-1224
	mmu-miR-467f	mghv-mir-M1-8*	mmu-miR-199a-3p/-199b	mmu-miR-741	mmu-miR-196a-2*
	mmu-miR-1982*	mmu-miR-1839-5p	mmu-miR-669f-5p	mmu-miR-761	mmu-miR-3081*
	mghv-miR-M1-7-3p	mmu-miR-1967	mmu-miR-344b*	mmu-miR-466o-3p	mmu-miR-3072
	mmu-miR-669b*	mmu-miR-466c-5p	mmu-miR-1935	mmu-miR-150	mmu-miR-34c
	mmu-let-7g	mmu-miR-99b	mmu-miR-208a-5p	mmu-miR-99b*	mmu-miR-322
	mmu-miR-125a-5p	mmu-miR-467g	mmu-miR-3082-5p	mmu-miR-106b	mmu-miR-27a*
	**mmu-miR-223**	mmu-miR-133a	mmu-miR-223*	mmu-miR-1899	mmu-miR-1896
	mmu-miR-1892	mmu-miR-468	mmu-miR-3474	mmu-miR-3072*	mmu-miR-379
	mmu-miR-465b-5p	mghv-mir-M1-2-5p	mmu-miR-344d-2*	mmu-miR-1192	mmu-miR-1982.1/1982.2
	mmu-miR-669a-3-3p	mmu-miR-328*	mmu-miR-466d-3p	mmu-miR-203	mmu-miR-423-5p
	mmu-miR-199b*	mmu-miR-221	mmu-miR-10b	mmu-miR-23b*	mmu-miR-1927
	mmu-miR-882	mmu-miR-491*	mmu-miR-30b	mmu-miR-181d	mmu-miR-466f
	mmu-miR-341	mmu-miR-378/378b	mmu-miR-669n	mmu-miR-1958	mmu-miR-93
	mmu-miR-125b-5p	mmu-miR-488	mmu-miR-34b-5p	mmu-miR-466j	mmu-miR-193b
	mmu-miR-710	mmu-miR-135a-1*	mmu-miR-742	mmu-miR-214*	mmu-miR-652
	mmu-miR-466a/p-5p	mmu-miR-712	mmu-miR-3102	mmu-miR-511-3p	mmu-miR-3084
	mmu-let-7i	mmu-miR-466d-5p	mmu-miR-874*	mmu-miR-148b*	mmu-miR-20a*
	mmu-miR-298	mmu-miR-546	mmu-miR-466e-5p	mmu-miR-200c*	mmu-miR-1957
	mmu-miR-1934	mmu-miR-143	mmu-miR-1193-3p	mmu-miR-365	mmu-miR-547*
	mmu-miR-1981*	mmu-miR-505-5p	mmu-miR-692	mmu-miR-331-5p	mmu-miR-669p*
	mmu-miR-669k*	mmu-miR-196b*	mmu-miR-669f-3p	mmu-miR-3101*	mmu-miR-484
	mmu-miR-669e*	mmu-miR-129-2-3p	mmu-miR-29c*	mmu-miR-381	mmu-miR-301b
	mmu-miR-206	mmu-miR-1a-2*	mmu-miR-431*	mmu-miR-194-1*	mmu-miR-344*
	mmu-miR-871-3p	mmu-miR-28*	mmu-miR-377*	mmu-miR-673-3p	mmu-miR-1839-3p
	mmu-miR-30d	mmu-miR-344c*	mmu-miR-139-5p	mmu-miR-539-3p	mmu-miR-669h-5p
	mmu-miR-1981	mmu-miR-760-3p	mmu-miR-544-5p	mmu-miR-374/374c	mghv-miR-M1-13*
	mmu-miR-155	mmu-miR-181a	mmu-miR-325	mmu-miR-31	mmu-miR-181b
	mmu-miR-669e	mmu-miR-466b/o-5p	mmu-miR-186*	mghv-miR-M1-3	mmu-miR-509-5p
	mmu-miR-23a	mmu-miR-199a-5p	mmu-miR-497	mmu-miR-669c*	mmu-miR-1247*
	mmu-miR-433	mmu-miR-881	mmu-miR-1188*	mmu-miR-410	mmu-miR-378*
	mmu-miR-425	**mmu-miR-210**	mmu-miR-758*	mmu-miR-106b*	mcmv-miR-m88-1*
	mmu-miR-463	mmu-miR-3069-3p	mmu-miR-615-3p	mmu-miR-671-3p	mmu-miR-128
	mmu-miR-669a/o-3p	mmu-miR-2136	mmu-miR-383*	mmu-miR-490-5p	mmu-miR-3084*
	mmu-miR-1929	mmu-miR-196a	mmu-miR-342-3p	mmu-miR-335-5p	mmu-miR-878-5p
	mmu-miR-30e	mmu-miR-499*	mmu-miR-3067*	mmu-miR-125b-2-3p	mmu-miR-205*
	mmu-miR-466a-5p	mmu-miR-320	mmu-miR-25*	mmu-miR-3102-3p.2	mmu-miR-204*
	mmu-let-7b	mmu-miR-30c	mmu-miR-500*	mmu-miR-219-3p	
	mmu-miR-362-3p	mmu-miR-455*	mmu-miR-3098-5p	mmu-miR-193	

DEmiRNAs in bold were confirmed using qRT-PCR. DEmiRNAs: differentially expressed microRNAs; LCSCs: lung cancer stem cells; BMHSCs: bone marrow hematopoietic stem cells; CTX: cyclophosphamide; SHSB: Shuanghuang Shengbai; ANOVA: analysis of variance; qRT-PCR: quantitative real time-polymerase chain reaction.

### Confirmation of 12 DEmiRNAs

**Figure 3:**
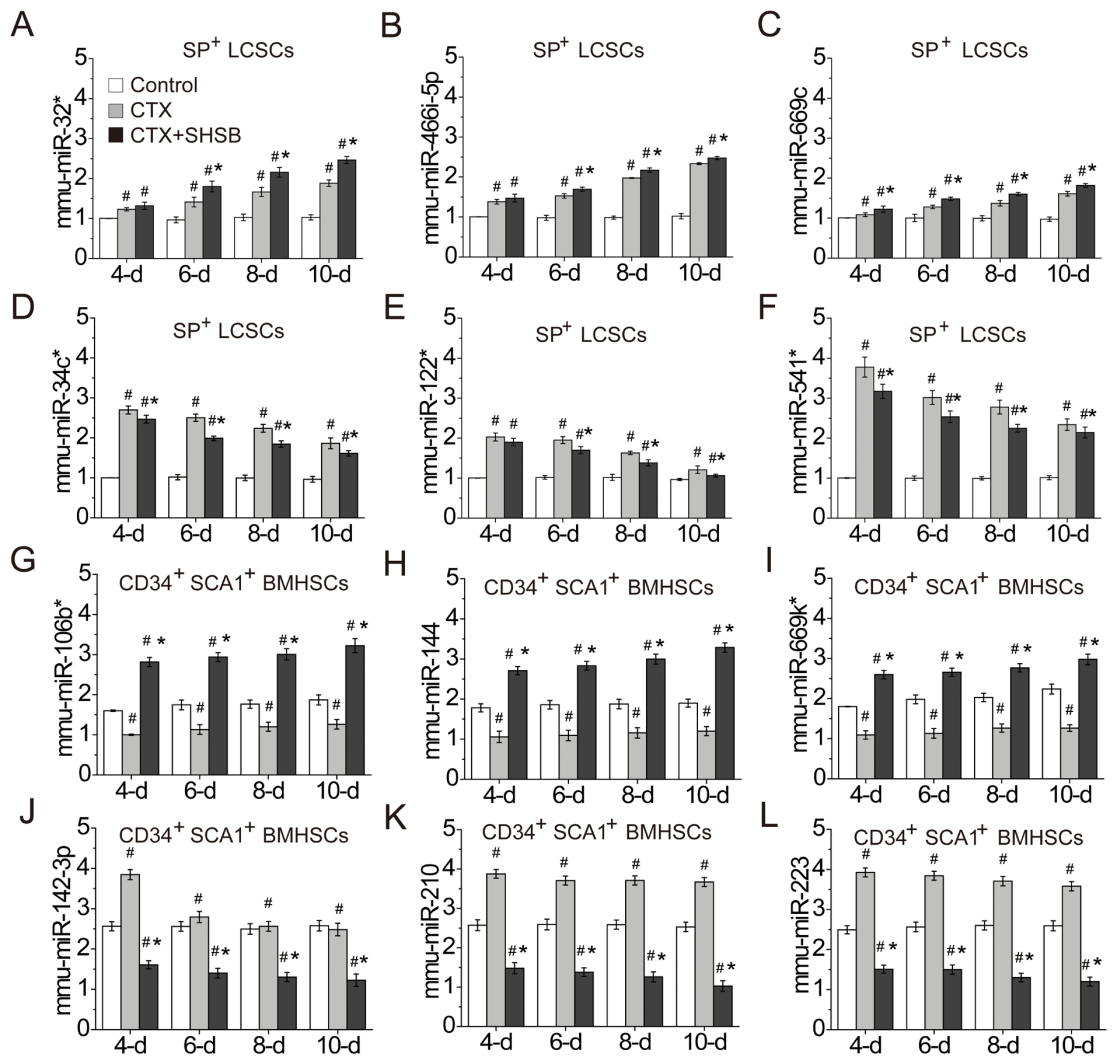
Confirmation of DEmiRNAs **(A)** mmu-miR-32* in SP^+^ LCSCs. **(B)** mmu-miR-466i-5p in SP^+^ LCSCs. **(C)** mmu-miR-669c in SP^+^ LCSCs. **(D)** mmu-miR-34c* in SP^+^ LCSCs. **(E)** mmu-miR-122* in SP^+^ LCSCs. **(F)** mmu-miR-541* in SP^+^ LCSCs. **(G)** mmu-miR-106b* in CD34^+^SCA1^+^ BMHSCs. **(H)** mmu-miR-144 in CD34^+^SCA1^+^ BMHSCs. **(I)** mmu-miR-669k* in CD34^+^SCA1^+^ BMHSCs. **(J)** mmu-miR-142-3p in CD34^+^SCA1^+^ BMHSCs. **(K)** mmu-miR-210 in CD34^+^SCA1^+^ BMHSCs. **(L)** mmu-miR-223 in CD34^+^SCA1^+^ BMHSCs. #: P<0.05 when compared with control group. *: P<0.05 when compared with CTX group. DEmiRNAs: differentially expressed microRNAs; LCSCs: lung cancer stem cells; BMHSCs: bone marrow hematopoietic stem cells; CTX: cyclophosphamide; SHSB: Shuanghuang Shengbai. Quantitative real time-polymerase chain reaction was performed to confirm the expressions of these microRNAs. For all these tests, N=6.

QRT-PCR was performed to confirm the differential expression of 12 DEmiRNAs (Table 2 and Figure 3). As shown in Figure 3, mmu-miR-32* (Figure 3A), mmu-miR-466i-5p (Figure 3B) and mmu-miR-669c (Figure 3C) in SP^+^ LCSCs were significantly up-regulated after CTX treatment. These microRNAs were further up-regulated after CTX+SHSB treatment. These results demonstrated that CTX promoted the expression of these microRNAs in SP^+^ LCSCs, and SHSB further strengthened their expression.

**Table 2:** Primers for miRNA qRT-PCR

miRNA	RT (Primer)	PCR (Forward primer)	PCR (Reverse primer)
mir-144	GTCGTATCCAGTGCAGGGTCCGAGG TATTCGCACTGGATACGACCAGTACA	CGGCCGGTACAGTATAGATGA	GTGCAGGGTCCGAGGT
mir-106b*	GTCGTATCCAGTGCAGGGTCCGAGG TATTCGCACTGGATACGACCGCAGCA	AATGCCGCACTGTGGGTACT	GTGCAGGGTCCGAGGT
mir-669k*	GTCGTATCCAGTGCAGGGTCCGAGG TATTCGCACTGGATACGACCGCACAC	GCCCGGTGTGCATGTGTGTATAGTT	GTGCAGGGTCCGAGGT
mir-142-3p	GTCGTATCCAGTGCAGGGTCCGAGG TATTCGCACTGGATACGACCTCCATA	GCGCTGTAGTGTTTCCTACTT	GTGCAGGGTCCGAGGT
mir-223	GTCGTATCCAGTGCAGGGTCCGAGG TATTCGCACTGGATACGACCTGGGGT	CCGCCCGTGTCAGTTTGTCAAAT	GTGCAGGGTCCGAGGT
mir-210	GTCGTATCCAGTGCAGGGTCCGAGG TATTCGCACTGGATACGACCTCAGCC	AATCCTGTGCGTGTGACAGC	GTGCAGGGTCCGAGGT
mir-32*	GTCGTATCCAGTGCAGGGTCCGAGG TATTCGCACTGGATACGACCAATATC	CGCGCGGCCAATTTAGTGTGTGT	GTGCAGGGTCCGAGGT
mir-669C	GTCGTATCCAGTGCAGGGTCCGAGG TATTCGCACTGGATACGACCACACAC	CGCGCCATAGTTGTGTGTGGAT	GTGCAGGGTCCGAGGT
mir-466i-5p	GTCGTATCCAGTGCAGGGTCCGAGG TATTCGCACTGGATACGACCCACACA	CGCCCTGTGTGTGTGTGTG	GTGCAGGGTCCGAGGT
mir-122*	GTCGTATCCAGTGCAGGGTCCGAGG TATTCGCACTGGATACGACCTTAGTG	CGCCCAAACGCCATTATCA	GTGCAGGGTCCGAGGT
mir-34C*	GTCGTATCCAGTGCAGGGTCCGAGG TATTCGCACTGGATACGACCCCTGGC	GCCCAATCACTAACCACACA	GTGCAGGGTCCGAGGT
mir-541*	GTCGTATCCAGTGCAGGGTCCGAGG TATTCGCACTGGATACGACCAGTATG	ACACCCTGGCGAACACAGAATC	GTGCAGGGTCCGAGGT
U6		CTCGCTTCGGCAGCACA	AACGCTTCACGAATTTGCGT

**MiRNA:** microRNA; qRT-PCR: quantitative real time-polymerase chain reaction; RT: reverse transcription; PCR: polymerase chain reaction.

In addition, mmu-miR-106b* (Figure 3G), mmu-miR-144 (Figure 3H) and mmu-miR-669k* (Figure 3I) in CD34^+^SCA1^+^ BMHSCs were down-regulated after CTX treatment. However, these microRNAs were up-regulated after CTX+SHSB treatment. On the contrary, mmu-miR-142-3p (Figure 3J), mmu-miR-210 (Figure 3K) and mmu-miR-223 (Figure 3L) in CD34^+^SCA1^+^ BMHSCs were up-regulated after CTX treatment. These microRNAs were down-regulated after CTX+SHSB treatment. These results indicated that SHSB reversed the effects of CTX on microRNAs like mmu-miR-106b*, mmu-miR-144, mmu-miR-669k*, mmu-miR-142-3p, mmu-miR-210 and mmu-miR-223 in CD34^+^SCA1^+^ BMHSCs.

## DISCUSSION

CTX frequently causes myelosuppression [5], and SHSB granule can cure myelosuppression without promoting the development of lung cancer [11, 12]. However, its mechanism and therapeutic targets are still unclear.

In this study, CTX treatment strongly suppressed tumor growth (Figure 1A). CTX decreased SP^+^ LCSCs ratio (Figure 1G) and CD133^+^ LCSCs ratio (Figure 1I). Reportedly, alkylating units in the CTX-derived phosmoramide mustard in human can alkylate DNA [20], and CTX induces cell apoptosis [21, 22]. Our results were consistent with the suppressive effects of CTX on lung cancer [4]. These results also suggested that CTX might suppress lung cancer growth via decreasing SP^+^ LCSCs and CD133^+^ LCSCs.

More importantly, proliferation index of lung cancer cells (Figure 1E), SP^+^ LCSCs ratio (Figure 1G) and CD24^+^IGF1R^+^ LCSCs ratio (Figure 1H) in CTX+SHSB group were remarkably lower than that in control and CTX groups. These results agreed well with the inhibitory effects of SHSB on the transformation of lung cancer cells from G0/G1 phase to S phase [10]. Also, these results indicated that SHSB granule might suppress the growth of lung cancer via decreasing SP^+^ LCSCs.

In the present study, CTX remarkably reduced the number of WBCs (Figure 1B). This result was consistent with the fact that CTX could cause myelosuppression [5]. After CTX treatment, declines were found in the proliferation index of BMCs (4-d; Figure 1F) and CD34^+^SCA1^+^ BMHSCs ratio (Figure 1J). This suggested that CTX might cause myelosuppression via inhibiting the proliferation of CD34^+^SCA1^+^ BMHSCs. In contrast, SHSB granule completely reversed the decrease in WBCs (Figure 1B). This agreed well with the fact that SHSB could cure myelosuppression [10-12]. Especially, SHSB remarkably increased the proliferation index of BMCs (Figure 1F) and CD34^+^SCA1^+^ BMHSCs ratio (Figure 1J). As WBCs derived from BMHSCs, our results indicated that SHSB might increase WBCs via promoting the proliferation of CD34^+^SCA1^+^ BMHSCs.

As shown in qRT-PCR results, CTX enhanced the expression of mmu-miR-32*, mmu-miR-466i-5p and mmu-miR-669c in SP^+^ LCSCs, and SHSB granule further strengthened their expression (Figure 3A–3C). Reportedly, miR-32 is located at 9q31.2 in a region of homozygous deletion in various types of cancer [23]. For instance, it promotes the growth, migration and invasion of colorectal carcinoma cells [24]. Currently, little is known about the role of miR-466i-5p in cancer development. Ionizing radiation can induce apoptosis in cochlea hair cells, and miR-466i-5p in HEI-OC1 cells is significantly up-regulated 12, 24 and 48 h after ionizing radiation [25]. MiR-669c is significantly down-regulated in mice with Bronchiolo-alveolar adenocarcinoma [26]. In this study, the up-regulations of miR-32*, miR-466i-5p and mmu-miR-669c in SP^+^ LCSCs after CTX treatment were consistent with previous studies [23, 25, 26]. Besides, these microRNAs were further up-regulated after CTX+SHSB treatment. This indicated that SHSB might further inhibit SP^+^ LCSCs proliferation via enhancing the expressions of miR-32*, miR-466i-5p and mmu-miR-669c.

Moreover, CTX significantly changed the expressions of mmu-miR-106b* (down-regulation), mmu-miR-144 (down-regulation), mmu-miR-669k* (down-regulation), mmu-miR-142-3p (up-regulation), mmu-miR-210 (up-regulation) and mmu-miR-223 (up-regulation) in CD34^+^SCA1^+^ BMHSCs (Figure 3G–3L). However, SHSB reversed the effects of CTX on these microRNAs (Figure 3G–3L). Among these microRNAs, little is known about the role of miR-106b*, mmu-miR-144 and miR-669k* in proliferation or differentiation of hematopoietic stem cells [27, 28]. In contrast, miR-142a-3p accelerates the formation and differentiation of hematopoietic stem cells [29]. MiR-210 level is increased in CD34^+^ cells in myelodysplastic syndromes, a disease caused by abnormal proliferation and differentiation of hematopoietic stem cells [30]. MiR-223 is preferentially expressed in bone marrow [31], and it induces human granulopoiesis [32]. Therefore, SHSB might enhance BMHSCs proliferation via up-regulating mmu-miR-106b*, mmu-miR-144 and mmu-miR-669k*, as well as down-regulating mmu-miR-142-3p, mmu-miR-210 and mmu-miR-223.

## MATERIALS AND METHODS

### Animals, cell line, and lung cancer models

Nude mice are immune deficient, and tumor can be easily established in them. Therefore, four-week old male BALB/C nude mice weighted 20±2g were purchased from Shanghai Laboratory Animal Center of Chinese Academy of Sciences [Batch Number: SCXK (Shanghai) 2007-0005]. These mice were bred in the Specific-Pathogen-Free animal laboratory of Longhua Hospital (Shanghai, China) under a condition of 24-26^o^C, 65-70% humidity and free access to diet and water.

Lung adenocarcinoma is one of the main kinds of lung cancer, and A549 is a cell line of human lung adenocarcinoma. In this study, A549 was obtained from the Shanghai Cell Bank of Chinese Academy of Sciences. A549 cells were cultured in F12k medium supplemented with 10% fetal bovine serum (Gibco, NY, USA). Then, 0.2 ml 1×10^7^/ml A549 cells with high proliferating activity were inoculated into the oxters of nude mice. Lung cancer models were successfully constructed four weeks after inoculation.

### Animal grouping and drug administration

The constructed cancer models were randomly divided into three groups, including control group (N=36), CTX group (i.e. CTX group; N=36), and CTX + SHSB granule group (i.e. CTX+SHSB group; N=36). Since CTX could lead to obvious myelosuppression, we utilized CTX to treat lung cancer to establish a mice model of myelosuppression. CTX was generated from Jiangsu Hengrui Medicine Co., Ltd (Batch Number: H32020857). Before usage, 1 mg/ml CTX was freshly prepared using physiological saline. SHSB granule was obtained from the drug preparation center of Longhua Hospital (Batch Number: Z05170773). SHSB granule was dissolved in double-distilled H_2_O to generate a 2 g/ml SHSB solution. Nude mice in control group were intraperitoneally injected with physiological saline (100 mg/kg/d) for three days, and these mice also took physiological saline (40 g/kg/d) via gastroenteric irrigation for 10 days. Nude mice in CTX group were intraperitoneally injected with CTX (100 mg/kg/d) for three days, and these mice also took physiological saline (40 g/kg/d) via gastroenteric irrigation for 10 days. Nude mice in CTX+SHSB group were intraperitoneally injected with CTX (100 mg/kg/d) for three days, and these mice also took SHSB (40 g/kg/d) via gastroenteric irrigation for 10 days. Intraperitoneal injection and gastroenteric irrigation were started at the same day for all mice. All the animal experiments were approved by the Animal Ethics Committee of Shanghai Longhua Hospital, China.

### General blood indexes and tumor mass

Eyeball was removed, and peripheral blood was taken from mice 4, 6, 8 or 10 days after intraperitoneal injection. WBCs, red blood cells and platelets were counted using an automatic blood cell analyzer (DxH 800™; Beckman Coulter Inc., Miami, FL, USA). Thereafter, mice were sacrificed by cutting off their neck under anesthesia. Xenograft tumors were obtained and weighted under a sterile and RNAase-free condition.

### Generation of lung cancer cells and BMCs

Lung cancer cells were generated from xenograft tumor (4-d, 6-d, 8-d and 10-d) after trypsin digestion. The femur and tibia were taken from the sacrificed mice (4-d, 6-d, 8-d and 10-d) and sterilized using 75% alcohol. BMCs were obtained after splitting and opening the femur and tibia. Then, 2 ml hemolysin was added into BMCs suspension to damage red blood cells. The suspension was centrifuged at 2000 rpm for 15 min.

### Determination of cell cycle and proliferation index

Lung cancer cells and BMCs were immobilized at 4°C for 10 h using 70% and absolute ethyl alcohol, respectively. Cells were washed with phosphate buffer solution. RNase A was added (final concentration: 50 μg/ml) to digest RNA at 37°C for 30 min. Propidium iodide solution was added (final concentration: 65 μg/ml) to stain cells for 30 min. Then, FACSCalibur™ Cell Sorting system (BD Biosciences; Franklin Lakes, NJ, USA) was utilized to perform flow cytometry to determine cell cycle. Based on cell cycle information, proliferation index was calculated using the following formula:

$$\text{Proliferation index}=\frac{S+G_{2}/M}{G_{0}/G_{1}+S+G_{2}/M}\times 100\%$$

In this formula, G_0_ /G_1_, S and G_2_ /M represent the relative numbers of cells in phase G_0_ /G_1_, S and G_2_ /M, respectively.

### Ratio of stem cells

For each sample of lung cancer cells (500 μl, 2×10^6^/ml), 10 μl Heochst 33342/Propidium iodide solution was added to mark SP^+^ LCSCs; 10 μl Anti-CD24 labeled with allophycocyanin and 20 μl Anti-IGF1R labeled with phycoerythrin were added to mark CD24^+^IGF1R^+^ LCSCs; 20 μl Anti-CD133 labeled with allophycocyanin was added to mark CD133^+^ LCSCs.

For each BMCs sample (500 μl, 2×10^6^/ml), 20 μl Anti-CD34 labeled with allophycocyanin and 20 μl Anti-SCA1 labeled with phycoerythrin were added to mark CD34^+^SCA1^+^ BMHSCs. Flow cytometry was used to detect the ratios of these stem cells.

### MicroRNA microarray analysis

Flow cytometry was used to isolate SP^+^ LCSCs and CD34^+^SCA1^+^ BMHSCs from lung cancer cells (8-d) and BMCs (6-d), respectively. For each sample, total RNA was isolated using Trizol Reagent (Invitrogen; CA, USA), and microRNA was purified using miRNeasy mini kit (Qiagen; Valencia, CA, USA). RNA quantity and quality were determined using NanoDrop spectrophotometer (ND-1000; Nano-Drop Technologies; DE, USA). Thereafter, the miRCURY™ Hy3™/Hy5™ Power labeling kit (Exiqon; Vedbaek, Denmark) was utilized to label 1 mg microRNA to miRCURY™ LNA Array (version: 16.0).

Microarray data were normalized using the Quantile method. Differentially expressed microRNAs (DEmiRNAs) between groups were identified via Volcano Plot filtering [criteria: Fold change ≥1.5 and P≤0.05] and analysis of variance (ANOVA; criterion: P≤0.05). Moreover, hierarchical clustering was conducted using the MeV software (v4.6; the institute for genomic research; http://www.tm4.org/mev.html) to determine the specificity of DEmiRNAs between groups.

### qRT-PCR

A total of 12 DEmiRNAs were randomly selected, and their relative levels were detected. Total RNA was isolated from SP^+^ LCSCs (4-d, 6-d, 8-d and 10-d) and CD34^+^SCA1^+^ BMHSCs (4-d, 6-d, 8-d and 10-d) using Trizol Reagent (Invitrogen). Reverse transcription was conducted in a 20 μl reaction system (condition: 37°C for 15 min and then 85°C for 5 s) using PrimeScript RT reagent kit (TaKaRa Biotechnology Co. Ltd.; Dalian, China). Thereafter, RT-PCR was performed in a 20 μl reaction system [condition: 95°C for 5 min, 40 cycle (95°C for 10 s, 60°C for 20 s, 72°C for 20 s, and 79°C for 20 s)] using the SYBR Premix Ex Taq kit (TaKaRa) and a PCR instrument (Rotor Gene 3000; Corbett Research; Sydney, Australia). Relative levels of these microRNAs were calculated using 2^-∆∆CT^ method and U6 levels.

### Statistical analysis

All data were shown in a “mean ± standard deviation” manner and analyzed using Statistical Product and Service Solutions (SPSS; version: 18.0) software. Differences between groups were determined using one-way ANOVA method. Least-Significant-Difference test was utilized under a condition of equal variance. Otherwise, Dunnett’s T3 method was used to adjust results. The threshold for statistical difference was set as P<0.05.

## CONCLUSION

To sum up, SHSB granule might cure CTX-induced myelosuppression and increase WBCs via enhancing CD34^+^SCA1^+^ BMHSCs proliferation (SHSB granule up-regulated the expressions of mmu-miR-106b*, mmu-miR-144 and mmu-miR-669k*, as well as down-regulated the expressions of mmu-miR-142-3p, mmu-miR-210 and mmu-miR-223 in CD34^+^SCA1^+^ BMHSCs). Besides, SHSB granule might also suppress lung cancer progression via inhibiting SP^+^ LCSCs proliferation (SHSB granule up-regulated miR-32*, miR-466i-5p and mmu-miR-669c in SP^+^ LCSCs). These findings might help us to better understand the mechanism of SHSB in curing myelosuppression and blocking lung cancer development. Our results might also provide potential therapeutic targets for treating CTX-caused myelosuppression in patients with lung cancer.

### Limitation

In the present study, microRNA profiling was only determined in SP^+^ LCSCs (8-d) and CD34^+^SCA1^+^ BMHSCs (6-d). A time-course microRNA profiling analysis might provide more information. Only the change patterns of 12 microRNAs were detected by using qRT-PCR. Much more microRNAs will be selected to perform qRT-PCR in our future study.

## SUPPLEMENTARY MATERIALS FIGURE


