# Supplementary figures and images for: Shuanghuang Shengbai granule cures myelosuppression and suppresses lung cancer progression: mechanism and therapeutic targets from the aspect of microRNAs
Source: Oncotarget. 2017 Jul 10;8(37):62154–66. doi: 10.18632/oncotarget.19129 (PMC5617494; doi:10.18632/oncotarget.19129)

## SUPPLEMENTARY MATERIALS

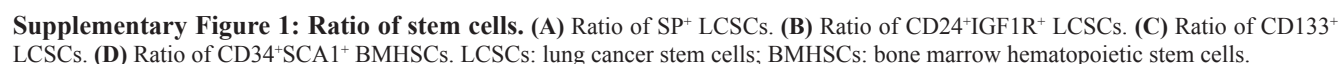

Supplement: Supplementary file 1 [file oncotarget-08-62154-s001.pdf]
